# 2-Amino-4-phenyl-4*H*,10*H*-1,3,5-triazino[1,2-*a*]benzimidazol-3-ium chloride

**DOI:** 10.1107/S1600536811013766

**Published:** 2011-04-16

**Authors:** Shaaban K. Mohamed, Mahmoud A. A. El-Remaily, Ahmed M. Soliman, Hossam Abdel-Ghany, Seik Weng Ng

**Affiliations:** a Division of Chemistry and Environmental Science, Manchester Metropolitan University, Manchester, England; b Department of Chemistry, Sohag University, Egypt; c Department of Chemistry, University of Malaya, 50603 Kuala Lumpur, Malaysia

## Abstract

2-Guanidinobenzimidazole condenses with benzaldehyde in the presence of hydro­chloric acid to form 2-amino-3,4-dihydro-4-phenyl-1,3,5-triazino[1,2-*a*]benzimidazole, which was isolated as its hydro­chloride, C_15_H_14_N_5_^+^·Cl^−^. The positive charge of the cation is formally placed on the double-bonded N atom of the dihydro­triazine ring. The six-membered dihydro­triazine that is fused with the benzimidazole ring system is relatively flat (r.m.s. deviation = 0.106 Å), with the methine C atom deviating most [0.164 (1) Å] from the mean-square plane. The phenyl ring connected to the methine C atom is disordered over two positions in a 0.558 (1):0.442 (1) ratio; the two orientations are aligned at 85.1 (1) and 89.6 (1)° with respect to the dihydro­triazine ring. In the crystal, adjacent cations and anions are linked by N—H⋯N and N—H⋯Cl hydrogen bonds, generating a double chain running along the *b* axis.

## Related literature

For the synthesis, see: Dolzhenko & Chui (2006 ▶); Martin *et al.* (1981 ▶); Nagarajan *et al.* (1970 ▶).
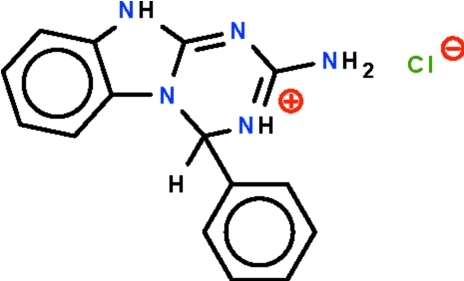


## Experimental

### 

#### Crystal data

C_15_H_14_N_5_^+^·Cl^−^
*M* *_r_* = 299.76
Triclinic, 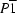

*a* = 8.6454 (5) Å
*b* = 9.0440 (4) Å
*c* = 9.7182 (6) Å
α = 83.306 (4)°
β = 70.956 (5)°
γ = 81.523 (4)°
*V* = 708.51 (7) Å^3^
*Z* = 2
Cu *K*α radiation
μ = 2.39 mm^−1^
*T* = 100 K
0.20 × 0.20 × 0.02 mm

#### Data collection

Agilent SuperNova Dual diffractometer with an Atlas detector
Absorption correction: multi-scan (*CrysAlis PRO*; Agilent, 2010 ▶) *T*_min_ = 0.647, *T*_max_ = 0.954
7924 measured reflections
2818 independent reflections
2667 reflections with *I* > 2σ(*I*)
*R*_int_ = 0.017

#### Refinement

*R*[*F* ^2^ > 2σ(*F* ^2^)] = 0.040
*wR*(*F* ^2^) = 0.106
*S* = 1.01
2818 reflections
237 parameters
6 restraints
H atoms treated by a mixture of independent and constrained refinement
Δρ_max_ = 0.33 e Å^−3^
Δρ_min_ = −0.29 e Å^−3^

### 

Data collection: *CrysAlis PRO* (Agilent, 2010 ▶); cell refinement: *CrysAlis PRO*; data reduction: *CrysAlis PRO*; program(s) used to solve structure: *SHELXS97* (Sheldrick, 2008 ▶); program(s) used to refine structure: *SHELXL97* (Sheldrick, 2008 ▶); molecular graphics: *X-SEED* (Barbour, 2001 ▶); software used to prepare material for publication: *publCIF* (Westrip, 2010 ▶).

## Supplementary Material

Crystal structure: contains datablocks global, I. DOI: 10.1107/S1600536811013766/bt5515sup1.cif

Structure factors: contains datablocks I. DOI: 10.1107/S1600536811013766/bt5515Isup2.hkl

Additional supplementary materials:  crystallographic information; 3D view; checkCIF report

## Figures and Tables

**Table 1:** Hydrogen-bond geometry (Å, °)

*D*—H⋯*A*	*D*—H	H⋯*A*	*D*⋯*A*	*D*—H⋯*A*
N1—H1⋯Cl1	0.88 (1)	2.23 (1)	3.1033 (16)	172 (2)
N4—H4⋯Cl1^i^	0.88 (1)	2.25 (1)	3.1060 (14)	165 (2)
N5—H3⋯N3^ii^	0.89 (1)	2.08 (1)	2.9643 (19)	176 (2)
N5—H2⋯Cl1^ii^	0.88 (1)	2.66 (2)	3.3147 (14)	132 (2)

Symmetry codes: (i) 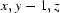; (ii) 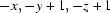.

**Fig. 1.**
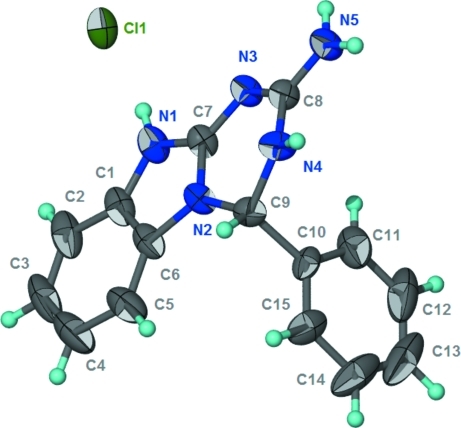
Anisotropic displacement ellipsoid plot (Barbour, 2001) of the C15H14N5 Cl salt at the 70% probability level; hydrogen atoms are drawn as spheres of arbitrary radius. The disorder is not shown.

## supplementary crystallographic information

### Comment

2-Guanidinobenzimidazole and aromatic aldehydes readily condense to form 2-amino-[1,3,5]triazino[1,2-*a*]benzimidazoles (Nagarajan *et al.*, 1970; Martin *et al.*, 1981); such compounds exhibit dihydrofolate reductase inhibitory activity (Dolzhenko & Chui, 2006). We added acetylacetone in the synthesis as the resulting compound possesses an amino subsitutent that is capable of further condensation, but the hydrochloric acid we used in the synthesis protonated the compound. The positive charge of the salt (Scheme I) is formally placed on the double-bonded N atom of the dihydrotriazine ring. The six-memebered dihydrotriazine that is fused with the benzimidazole ring-system is relatively flat, with the methine C deviating most from the mean-square plane. The phenyl ring that is connected to the methine C atom is disordered over two positions; the two orientations are aligned at nearly 90 ° with respect to the dihydrotriazine ring (Fig. 1). Adjacent cations and anions are linked by N–H···N and N–H···Cl hydrogen bonds to generate a linear chain motif (Table 1).

### Experimental

2-Guanidinobenzimidazole (1*H*-benzo[*d*]imidazol-2-yliminomethanediamine) (10 mmol), benzaldehyde (10 mmol) and excess of cyclohexanone (approx. 10 ml) along with few drops of concentrated hydrochloric acid was heated in *N*,*N*-dimethylformamide (50 ml) for 30 minutes. The product was collected and recrystallized from ethanol; m.p. 623 K.

### Refinement

Carbon-bound H-atoms were placed in calculated positions [C—H 0.95 to 1.00 Å, *U*_iso_(H) 1.2*U*_eq_(C)] and were included in the refinement in the riding model approximation.

The amino H-atoms were located in a difference Fourier map, and were refined with distance restraints of N–H 0.88±0.01 Å; their displacement parameters were refined.

The phenyl ring is disordered over two positions, and was refined as a rigid hexagon of 1.39 Å; the two *C*–C_phenyl_ distances were restrained to 1.50±0.01 Å. The disorder refined to a 55.8 (1):44.2 (1) ratio.

### Crystal data

**Table:**

C_15_H_14_N_5_^+^·Cl^−^	*Z* = 2
*M**_r_* = 299.76	*F*(000) = 312
Triclinic, *P*1	*D*_x_ = 1.405 Mg m^−^^3^
Hall symbol: -P 1	Cu *K*α radiation, λ = 1.54184 Å
*a* = 8.6454 (5) Å	Cell parameters from 4762 reflections
*b* = 9.0440 (4) Å	θ = 4.8–74.3°
*c* = 9.7182 (6) Å	µ = 2.39 mm^−^^1^
α = 83.306 (4)°	*T* = 100 K
β = 70.956 (5)°	Plate, colorless
γ = 81.523 (4)°	0.20 × 0.20 × 0.02 mm
*V* = 708.51 (7) Å^3^	

### Data collection

**Table:**

Agilent SuperNova Dual diffractometer with an Atlas detector	2818 independent reflections
Radiation source: SuperNova (Cu) X-ray Source	2667 reflections with *I* > 2σ(*I*)
Mirror	*R*_int_ = 0.017
Detector resolution: 10.4041 pixels mm^-1^	θ_max_ = 74.5°, θ_min_ = 4.8°
ω scans	*h* = −10→7
Absorption correction: multi-scan (*CrysAlis PRO*; Agilent, 2010)	*k* = −11→11
*T*_min_ = 0.647, *T*_max_ = 0.954	*l* = −12→11
7924 measured reflections	

### Refinement

**Table:**

Refinement on *F*^2^	Primary atom site location: structure-invariant direct methods
Least-squares matrix: full	Secondary atom site location: difference Fourier map
*R*[*F*^2^ > 2σ(*F*^2^)] = 0.040	Hydrogen site location: inferred from neighbouring sites
*wR*(*F*^2^) = 0.106	H atoms treated by a mixture of independent and constrained refinement
*S* = 1.01	*w* = 1/[σ^2^(*F*_o_^2^) + (0.0582*P*)^2^ + 0.4089*P*] where *P* = (*F*_o_^2^ + 2*F*_c_^2^)/3
2818 reflections	(Δ/σ)_max_ = 0.001
237 parameters	Δρ_max_ = 0.33 e Å^−^^3^
6 restraints	Δρ_min_ = −0.29 e Å^−^^3^

### Fractional atomic coordinates and isotropic or equivalent isotropic displacement parameters (Å^2^)

**Table:**

	*x*	*y*	*z*	*U*_iso_*/*U*_eq_	Occ. (<1)
Cl1	0.25096 (5)	0.91819 (4)	0.47166 (4)	0.03322 (14)	
N1	0.33424 (18)	0.61809 (16)	0.63707 (17)	0.0319 (3)	
N2	0.37251 (16)	0.37732 (15)	0.69693 (15)	0.0279 (3)	
N3	0.16136 (17)	0.46368 (14)	0.58756 (15)	0.0274 (3)	
N4	0.25040 (18)	0.20531 (15)	0.61885 (15)	0.0297 (3)	
N5	0.03987 (18)	0.28598 (15)	0.52257 (16)	0.0305 (3)	
C1	0.4578 (2)	0.5963 (2)	0.70333 (19)	0.0341 (4)	
C2	0.5488 (2)	0.6965 (3)	0.7323 (2)	0.0464 (5)	
H2A	0.5332	0.8008	0.7061	0.056*	
C3	0.6639 (3)	0.6360 (3)	0.8014 (2)	0.0587 (7)	
H3A	0.7280	0.7009	0.8236	0.070*	
C4	0.6878 (3)	0.4836 (3)	0.8389 (3)	0.0588 (7)	
H4A	0.7683	0.4470	0.8856	0.071*	
C5	0.5980 (2)	0.3834 (3)	0.8102 (2)	0.0465 (5)	
H5	0.6144	0.2790	0.8359	0.056*	
C6	0.4827 (2)	0.4437 (2)	0.74177 (19)	0.0330 (4)	
C7	0.28265 (19)	0.48468 (17)	0.63639 (17)	0.0269 (3)	
C8	0.1501 (2)	0.31762 (17)	0.57848 (17)	0.0263 (3)	
C9	0.3386 (2)	0.22131 (18)	0.71937 (17)	0.0281 (3)	
H9	0.4466	0.1566	0.6891	0.034*	0.558 (7)
H9'	0.4436	0.1519	0.6980	0.034*	0.442 (7)
C10	0.2434 (3)	0.1680 (4)	0.8782 (2)	0.0294 (11)	0.558 (7)
C11	0.0806 (3)	0.2263 (7)	0.9423 (3)	0.0475 (12)	0.558 (7)
H11	0.0253	0.2947	0.8871	0.057*	0.558 (7)
C12	−0.0012 (4)	0.1844 (7)	1.0873 (3)	0.0679 (19)	0.558 (7)
H12	−0.1124	0.2242	1.1311	0.081*	0.558 (7)
C13	0.0798 (7)	0.0843 (5)	1.1681 (2)	0.075 (2)	0.558 (7)
H13	0.0239	0.0557	1.2671	0.090*	0.558 (7)
C14	0.2426 (7)	0.0261 (3)	1.1039 (3)	0.0643 (19)	0.558 (7)
H14	0.2979	−0.0423	1.1591	0.077*	0.558 (7)
C15	0.3244 (5)	0.0679 (3)	0.9590 (3)	0.0408 (12)	0.558 (7)
H15	0.4356	0.0281	0.9151	0.049*	0.558 (7)
C10'	0.2393 (3)	0.1996 (4)	0.8737 (3)	0.0296 (14)	0.442 (7)
C11'	0.1106 (5)	0.3042 (4)	0.9424 (3)	0.0293 (11)	0.442 (7)
H11'	0.0941	0.4001	0.8945	0.035*	0.442 (7)
C12'	0.0060 (4)	0.2685 (4)	1.0813 (3)	0.0397 (13)	0.442 (7)
H12'	−0.0820	0.3400	1.1283	0.048*	0.442 (7)
C13'	0.0302 (4)	0.1282 (4)	1.1515 (3)	0.0356 (13)	0.442 (7)
H13'	−0.0413	0.1038	1.2464	0.043*	0.442 (7)
C14'	0.1589 (5)	0.0236 (3)	1.0827 (5)	0.0375 (12)	0.442 (7)
H14'	0.1755	−0.0723	1.1307	0.045*	0.442 (7)
C15'	0.2635 (4)	0.0592 (3)	0.9438 (4)	0.0342 (12)	0.442 (7)
H15'	0.3515	−0.0123	0.8968	0.041*	0.442 (7)
H1	0.300 (3)	0.7021 (17)	0.594 (2)	0.049 (6)*	
H4	0.251 (3)	0.1148 (14)	0.593 (2)	0.042 (6)*	
H2	0.020 (3)	0.1933 (13)	0.521 (2)	0.040 (5)*	
H3	−0.016 (2)	0.3623 (18)	0.486 (2)	0.043 (6)*	

### Atomic displacement parameters (Å^2^)

**Table:**

	*U*^11^	*U*^22^	*U*^33^	*U*^12^	*U*^13^	*U*^23^
Cl1	0.0303 (2)	0.0217 (2)	0.0453 (3)	−0.00372 (15)	−0.00500 (17)	−0.01220 (16)
N1	0.0319 (7)	0.0235 (7)	0.0438 (8)	−0.0042 (6)	−0.0128 (6)	−0.0120 (6)
N2	0.0253 (7)	0.0266 (7)	0.0345 (7)	0.0015 (5)	−0.0125 (6)	−0.0097 (5)
N3	0.0302 (7)	0.0172 (6)	0.0394 (7)	−0.0005 (5)	−0.0166 (6)	−0.0060 (5)
N4	0.0415 (8)	0.0186 (6)	0.0338 (7)	0.0019 (6)	−0.0192 (6)	−0.0065 (5)
N5	0.0364 (8)	0.0192 (6)	0.0432 (8)	−0.0051 (6)	−0.0213 (6)	−0.0033 (6)
C1	0.0256 (8)	0.0413 (9)	0.0361 (9)	−0.0060 (7)	−0.0040 (7)	−0.0195 (7)
C2	0.0350 (10)	0.0598 (13)	0.0451 (11)	−0.0182 (9)	0.0006 (8)	−0.0293 (9)
C3	0.0324 (10)	0.100 (2)	0.0505 (12)	−0.0253 (11)	−0.0044 (9)	−0.0371 (13)
C4	0.0336 (11)	0.097 (2)	0.0547 (13)	−0.0087 (11)	−0.0189 (10)	−0.0272 (13)
C5	0.0307 (9)	0.0688 (14)	0.0447 (10)	0.0017 (9)	−0.0170 (8)	−0.0173 (10)
C6	0.0228 (8)	0.0433 (10)	0.0345 (8)	−0.0015 (7)	−0.0071 (7)	−0.0174 (7)
C7	0.0258 (8)	0.0219 (7)	0.0335 (8)	0.0003 (6)	−0.0089 (6)	−0.0099 (6)
C8	0.0304 (8)	0.0191 (7)	0.0308 (8)	−0.0016 (6)	−0.0113 (6)	−0.0038 (6)
C9	0.0282 (8)	0.0274 (8)	0.0285 (8)	0.0023 (6)	−0.0099 (6)	−0.0052 (6)
C10	0.038 (3)	0.031 (2)	0.018 (2)	−0.0146 (15)	−0.0014 (18)	−0.0055 (13)
C11	0.0361 (19)	0.069 (4)	0.0365 (19)	−0.0140 (19)	−0.0019 (15)	−0.0186 (19)
C12	0.064 (3)	0.096 (6)	0.041 (3)	−0.050 (4)	0.010 (2)	−0.018 (3)
C13	0.126 (6)	0.074 (4)	0.028 (2)	−0.071 (4)	−0.003 (3)	−0.003 (3)
C14	0.131 (6)	0.038 (2)	0.0330 (19)	−0.045 (3)	−0.026 (3)	0.0075 (16)
C15	0.073 (3)	0.0228 (16)	0.0333 (17)	−0.0187 (19)	−0.021 (2)	0.0012 (13)
C10'	0.028 (3)	0.027 (2)	0.042 (3)	−0.0030 (17)	−0.019 (3)	−0.0078 (18)
C11'	0.032 (2)	0.029 (2)	0.0245 (18)	0.0022 (18)	−0.0072 (15)	−0.0058 (15)
C12'	0.035 (2)	0.042 (3)	0.035 (2)	0.000 (2)	−0.0032 (18)	−0.007 (2)
C13'	0.032 (2)	0.038 (3)	0.032 (3)	−0.009 (2)	−0.002 (2)	−0.001 (2)
C14'	0.035 (3)	0.029 (2)	0.045 (3)	−0.0102 (18)	−0.010 (2)	0.0078 (19)
C15'	0.027 (2)	0.031 (2)	0.043 (3)	−0.0072 (18)	−0.0083 (19)	0.0000 (19)

### Geometric parameters (Å, °)

**Table:**

N1—C7	1.348 (2)	C9—C10	1.551 (2)
N1—C1	1.397 (2)	C9—H9	1.0000
N1—H1	0.88 (1)	C9—H9'	1.0000
N2—C7	1.355 (2)	C10—C11	1.3900
N2—C6	1.400 (2)	C10—C15	1.3900
N2—C9	1.460 (2)	C11—C12	1.3900
N3—C7	1.329 (2)	C11—H11	0.9500
N3—C8	1.3538 (19)	C12—C13	1.3900
N4—C8	1.344 (2)	C12—H12	0.9500
N4—C9	1.452 (2)	C13—C14	1.3900
N4—H4	0.882 (10)	C13—H13	0.9500
N5—C8	1.319 (2)	C14—C15	1.3900
N5—H2	0.88 (1)	C14—H14	0.9500
N5—H3	0.89 (1)	C15—H15	0.9500
C1—C2	1.390 (2)	C10'—C11'	1.3900
C1—C6	1.391 (3)	C10'—C15'	1.3900
C2—C3	1.389 (3)	C11'—C12'	1.3900
C2—H2A	0.9500	C11'—H11'	0.9500
C3—C4	1.387 (4)	C12'—C13'	1.3900
C3—H3A	0.9500	C12'—H12'	0.9500
C4—C5	1.382 (3)	C13'—C14'	1.3900
C4—H4A	0.9500	C13'—H13'	0.9500
C5—C6	1.386 (3)	C14'—C15'	1.3900
C5—H5	0.9500	C14'—H14'	0.9500
C9—C10'	1.471 (3)	C15'—H15'	0.9500
			
C7—N1—C1	108.78 (14)	N2—C9—H9	107.9
C7—N1—H1	123.8 (16)	C10'—C9—H9	115.7
C1—N1—H1	127.2 (16)	C10—C9—H9	107.9
C7—N2—C6	109.45 (14)	N4—C9—H9'	110.6
C7—N2—C9	121.34 (13)	N2—C9—H9'	110.7
C6—N2—C9	128.97 (14)	C10'—C9—H9'	110.6
C7—N3—C8	113.66 (13)	C10—C9—H9'	102.7
C8—N4—C9	123.13 (13)	C11—C10—C15	120.0
C8—N4—H4	117.9 (14)	C11—C10—C9	120.27 (17)
C9—N4—H4	118.3 (14)	C15—C10—C9	119.63 (17)
C8—N5—H2	122.4 (14)	C12—C11—C10	120.0
C8—N5—H3	117.3 (14)	C12—C11—H11	120.0
H2—N5—H3	120 (2)	C10—C11—H11	120.0
C2—C1—C6	121.02 (19)	C11—C12—C13	120.0
C2—C1—N1	131.53 (19)	C11—C12—H12	120.0
C6—C1—N1	107.46 (14)	C13—C12—H12	120.0
C1—C2—C3	116.5 (2)	C14—C13—C12	120.0
C1—C2—H2A	121.7	C14—C13—H13	120.0
C3—C2—H2A	121.7	C12—C13—H13	120.0
C4—C3—C2	121.9 (2)	C15—C14—C13	120.0
C4—C3—H3A	119.1	C15—C14—H14	120.0
C2—C3—H3A	119.1	C13—C14—H14	120.0
C5—C4—C3	121.9 (2)	C14—C15—C10	120.0
C5—C4—H4A	119.1	C14—C15—H15	120.0
C3—C4—H4A	119.1	C10—C15—H15	120.0
C4—C5—C6	116.2 (2)	C11'—C10'—C15'	120.0
C4—C5—H5	121.9	C11'—C10'—C9	122.6 (2)
C6—C5—H5	121.9	C15'—C10'—C9	116.8 (2)
C1—C6—C5	122.43 (18)	C12'—C11'—C10'	120.0
C1—C6—N2	105.86 (15)	C12'—C11'—H11'	120.0
C5—C6—N2	131.71 (18)	C10'—C11'—H11'	120.0
N3—C7—N1	125.39 (15)	C11'—C12'—C13'	120.0
N3—C7—N2	126.15 (14)	C11'—C12'—H12'	120.0
N1—C7—N2	108.44 (14)	C13'—C12'—H12'	120.0
N5—C8—N4	119.28 (14)	C14'—C13'—C12'	120.0
N5—C8—N3	118.04 (14)	C14'—C13'—H13'	120.0
N4—C8—N3	122.63 (14)	C12'—C13'—H13'	120.0
N4—C9—N2	105.74 (12)	C15'—C14'—C13'	120.0
N4—C9—C10'	113.24 (18)	C15'—C14'—H14'	120.0
N2—C9—C10'	105.86 (18)	C13'—C14'—H14'	120.0
N4—C9—C10	111.64 (16)	C14'—C15'—C10'	120.0
N2—C9—C10	115.56 (18)	C14'—C15'—H15'	120.0
N4—C9—H9	107.9	C10'—C15'—H15'	120.0
			
C7—N1—C1—C2	−179.19 (18)	C6—N2—C9—N4	−164.88 (15)
C7—N1—C1—C6	0.91 (19)	C7—N2—C9—C10'	−99.0 (2)
C6—C1—C2—C3	−0.3 (3)	C6—N2—C9—C10'	74.7 (2)
N1—C1—C2—C3	179.83 (18)	C7—N2—C9—C10	−102.57 (19)
C1—C2—C3—C4	0.4 (3)	C6—N2—C9—C10	71.1 (2)
C2—C3—C4—C5	−0.3 (3)	N4—C9—C10—C11	−53.8 (2)
C3—C4—C5—C6	0.0 (3)	N2—C9—C10—C11	67.1 (2)
C2—C1—C6—C5	0.0 (3)	C10'—C9—C10—C11	47.2 (10)
N1—C1—C6—C5	179.93 (16)	N4—C9—C10—C15	129.8 (2)
C2—C1—C6—N2	−179.84 (15)	N2—C9—C10—C15	−109.3 (3)
N1—C1—C6—N2	0.07 (18)	C10'—C9—C10—C15	−129.2 (11)
C4—C5—C6—C1	0.1 (3)	C15—C10—C11—C12	0.0
C4—C5—C6—N2	179.93 (18)	C9—C10—C11—C12	−176.4 (3)
C7—N2—C6—C1	−1.03 (18)	C10—C11—C12—C13	0.0
C9—N2—C6—C1	−175.29 (15)	C11—C12—C13—C14	0.0
C7—N2—C6—C5	179.13 (18)	C12—C13—C14—C15	0.0
C9—N2—C6—C5	4.9 (3)	C13—C14—C15—C10	0.0
C8—N3—C7—N1	170.54 (15)	C11—C10—C15—C14	0.0
C8—N3—C7—N2	−11.2 (2)	C9—C10—C15—C14	176.4 (3)
C1—N1—C7—N3	176.97 (15)	N4—C9—C10'—C11'	−72.3 (3)
C1—N1—C7—N2	−1.56 (18)	N2—C9—C10'—C11'	43.1 (3)
C6—N2—C7—N3	−176.89 (15)	C10—C9—C10'—C11'	−155.5 (11)
C9—N2—C7—N3	−2.1 (2)	N4—C9—C10'—C15'	98.6 (3)
C6—N2—C7—N1	1.62 (18)	N2—C9—C10'—C15'	−146.0 (3)
C9—N2—C7—N1	176.39 (13)	C10—C9—C10'—C15'	15.5 (9)
C9—N4—C8—N5	−160.68 (15)	C15'—C10'—C11'—C12'	0.0
C9—N4—C8—N3	21.9 (2)	C9—C10'—C11'—C12'	170.6 (3)
C7—N3—C8—N5	−175.88 (15)	C10'—C11'—C12'—C13'	0.0
C7—N3—C8—N4	1.6 (2)	C11'—C12'—C13'—C14'	0.0
C8—N4—C9—N2	−31.2 (2)	C12'—C13'—C14'—C15'	0.0
C8—N4—C9—C10'	84.3 (2)	C13'—C14'—C15'—C10'	0.0
C8—N4—C9—C10	95.3 (2)	C11'—C10'—C15'—C14'	0.0
C7—N2—C9—N4	21.46 (19)	C9—C10'—C15'—C14'	−171.2 (3)

### Hydrogen-bond geometry (Å, °)

**Table:**

*D*—H···*A*	*D*—H	H···*A*	*D*···*A*	*D*—H···*A*
N1—H1···Cl1	0.88 (1)	2.23 (1)	3.1033 (16)	172 (2)
N4—H4···Cl1^i^	0.88 (1)	2.25 (1)	3.1060 (14)	165 (2)
N5—H3···N3^ii^	0.89 (1)	2.08 (1)	2.9643 (19)	176 (2)
N5—H2···Cl1^ii^	0.88 (1)	2.66 (2)	3.3147 (14)	132 (2)

Symmetry codes: (i) *x*, *y*−1, *z*; (ii) −*x*, −*y*+1, −*z*+1.
